# Genome-Wide Association Analysis of Effective Tillers in Rice under Different Nitrogen Gradients

**DOI:** 10.3390/ijms25052969

**Published:** 2024-03-04

**Authors:** Yuzhuo Liu, Wei Xin, Liqiang Chen, Yuqi Liu, Xue Wang, Cheng Ma, Laiyuan Zhai, Yingying Feng, Jiping Gao, Wenzhong Zhang

**Affiliations:** 1 College of Agriculture, Shenyang Agricultural University, Shenyang 110866, China; 2023200066@stu.syau.edu.cn (Y.L.); 2019200059@stu.syau.edu.cn (L.C.); 2023200065@stu.syau.edu.cn (Y.L.); wangxue1695@163.com (X.W.); 2021220233@stu.syau.edu.cn (C.M.); fengyingying7576@163.com (Y.F.); 2 College of Agriculture, Northeast Agricultural University, Harbin 150030, China; xinweineau@163.com; 3 Shenzhen Branch, Guangdong Laboratory for Lingnan Modern Agriculture, Agricultural Genomics Institute at Shenzhen, Chinese Academy of Agricultural Sciences, Shenzhen 518120, China; zhailaiyuan@caas.cn

**Keywords:** rice, tillering, nitrogen, genome-wide association studies

## Abstract

Nitrogen is a crucial element that impacts rice yields, and effective tillering is a significant agronomic characteristic that can influence rice yields. The way that reduced nitrogen affects effective tillering is a complex quantitative trait that is controlled by multiple genes, and its genetic basis requires further exploration. In this study, 469 germplasm varieties were used for a genome-wide association analysis aiming to detect quantitative trait loci (QTL) associated with effective tillering at low (60 kg/hm^2^) and high (180 kg/hm^2^) nitrogen levels. QTLs detected over multiple years or under different treatments were scrutinized in this study, and candidate genes were identified through haplotype analysis and spatio-temporal expression patterns. A total of seven genes (*NAL1*, *OsCKX9*, *Os01g0690800*, *Os02g0550300*, *Os02g0550700*, *Os04g0615700*, and *Os04g06163000*) were pinpointed in these QTL regions, and were considered the most likely candidate genes. These results provide favorable information for the use of auxiliary marker selection in controlling effective tillering in rice for improved yields.

## 1. Introduction

Rice is one of the world’s most important food crops. For example, in 2018, it was the primary source of nutrition for more than 50% of the world’s population [1], and rice production accounted for 32.24% of China’s total grain production (http://www.stats.gov.cn/, accessed on 2 November 2022). As the world’s population continues to increase, the area of arable land is gradually decreasing. Thus, providing higher rice yields is a top priority in the context of meeting the food demands of an increasing population [2]. Ensuring high rice yields while reducing nitrogen fertilizer application is a crucial challenge for rice breeding researchers. In recent years, significant progress has been made in efficiently utilizing nitrogen in rice. Several genes, such as *DEP1* [3], *NRT1.1B* [4,5], *GRF4* [6], *NGR5* [7], and *Nhd1* [8], have been cloned and identified. These genes are expected to greatly enhance both yield and nitrogen utilization in rice. Thus, prioritizing higher rice yields is essential to meet the food demands of a growing population. Tiller production and development in rice are closely related to the presence of nitrogen. However, excessive nitrogen fertilizer application makes tillers compete with main branches for resources, leading to excessive tillering, which in turn reduces grain yield and lowers the nitrogen uptake rate [9]. The quantity of effective tillers directly influences the number of spikes per plant, subsequently affecting rice yield. Rice tillering is regulated by multiple genes. For instance, the first cloned rice tiller-related gene, *MOC1* [10], stimulates tiller elongation while preventing tiller shoot formation and axillary meristem initiation. Solanum lactones (SLs) are a category of terpenoid phytohormones that inhibit the growth of tiller shoots in rice [11]. Mutations in *HTD3* result in the upregulation of several solanum lactone and abscisic acid-related genes, leading to a moderate increase in rice tillering. Typical SL mutants exhibit dwarfism and increased tillering. The most critical factors for SL synthesis are *HTD1/D17* [12], *D10* [13], and *D27*) [14], which are responsible for SL synthesis, and *HTD2*/*D14* [15] and *D53* [16], which are responsible for SL signaling. Previous studies have shown increased tiller numbers and yields by increasing nitrogen uptake, while *OsNLP4-OsNiR* [17] increases tiller number and output by enhancing nitrogen assimilation and nitrogen use efficiency (NUE). Furthermore, nitrogen-induced *OsmiR393* [18] attenuates the sensitivity of axillary buds to growth hormones while promoting rice tillering. Four significant low nitrogen-induced growth response agronomic traits have been investigated, including plant height, tiller number, chlorophyll content, and leaf length [19]. It has also been found that *OsNR2* increased effective tiller number, seed yield, and NUE in japonica rice, while also enhancing its effect via interactions with additional infiltrated indica *OsNRT1.1B* alleles [20].

With the achievements of the Rice Genome Project, additional genes and quantitative trait loci (QTL) have been discovered [21]. Genome-wide association studies (GWAS) provide an effective method for identifying QTL associated with complex agronomic traits. GWAS has previously been successfully applied to many other plant species, including *Arabidopsis* [22,23], maize [24], and cotton [25]. For the genetic dissection of agronomic traits in rice, GWAS has identified genes controlling grain type, including the *OsSNB* gene [26], while *OsSPL18* has been shown to control rice grain size [27]. Furthermore, *D88*/*D14* [28] regulates branching and affects spike structure. There are many relevant reports on the response of rice to nitrogen. For example, the *Ghd7* transcription factor represses *ARE1* expression while improving nitrogen utilization and seed yield in rice [29]. However, genes involved in the response of rice tillers to low nitrogen remain relatively unexplored, and the genetic basis and molecular mechanism of the reaction of rice tillers to nitrogen have not yet been clarified.

In this study, 469 germplasm resources (including 267 japonica rice and 202 indica rice) from the Rice SNP-Seek Database (https://snp-seek.irri.org/, accessed on 4 May 2020) were used to determine the effective tillers of rice at different nitrogen levels in 2021 and 2022. Genome-wide association analysis was employed to identify the QTL associated with effective tillers of rice under different nitrogen levels. The candidate genes were then refined through haplotype analysis and gene function annotation. These results provide essential genomic resources for rice breeding and establish a solid foundation for investigating the genetic basis of achieving synergistic high yield and quality of rice with reduced nitrogen fertilizer application.

## 2. Results

### 2.1. Phenotypic Variation

**Figure 1:**
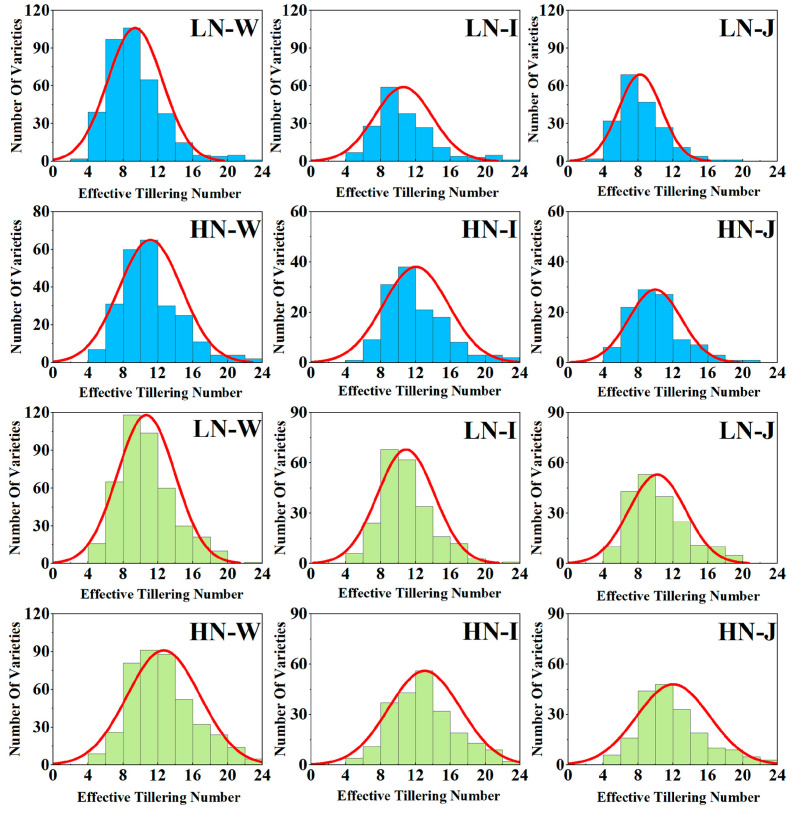
ETN frequency distribution of 469 germplasm resources. Blue and green represent 2021 and 2022 respectively. LN-W: The whole population under low nitrogen; LN-I: The indica population under low nitrogen; LN-J: The japonica population under low nitrogen; HN-W: The whole population under high nitrogen; HN-I: The indica population under high nitrogen; HN-J: The japonica population under high nitrogen.

To assess the phenotypic variation of 469 rice varieties under low and high nitrogen conditions, the frequency distribution statistics of effective tillers were determined over two years (2021 and 2022) for the whole, indica, and japonica populations. The phenotypes of each population exhibited continuous distribution, consistent with the genetic characteristics of quantitative traits controlled by multiple genes (Figure 1, Table S5).

Compared to that of the high nitrogen conditions, the rice ETN was observed to be decreased under low nitrogen conditions, and the indica subpopulation ETN was higher than that of the japonica subpopulation for both years under both nitrogen conditions. ETN had coefficients of variation ranging from 29.06% to 32.30% under low nitrogen conditions, compared to 29.00% to 35.15% under high nitrogen conditions (Table 1).

### 2.2. SNP Validation and Population Structure Analysis

A total of 2,801,841 high-quality SNPs were obtained, with a missing rate lower than 20% and a minor allele frequency (MAF) of >5%. An ultra-dense SNP genetic map was constructed based on the distribution of SNPs on chromosomes (Figure S1). These SNP markers were densely and evenly distributed across the rice genome, covering a total length of 373.05 Mb. The average distance between two adjacent SNPs on the 12 chromosomes was 133.96 bp. Among these, the average distance between SNPs on chromosome 5 was 162.75 bp, and on chromosome 10, it was 108.13 bp. Chromosome 1 had the longest length of 43.25 Mb, with 339,631 labeled SNPs, while chromosome 9 had the shortest length of 22.95 Mb and the least number of SNPs, with 162,135 SNPs (Table S2).

**Figure 2:**
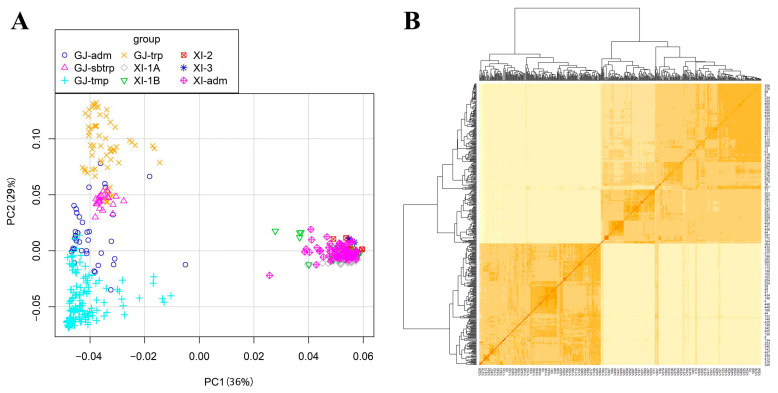
The population structure and the kinship of 469 rice. (**A**) Principal component analysis (PCA) plots for the first two components of 469 rice accessions. Each color represents a different population: Four populations of japonica (*GJ-adm*, *GJ-sbtrp*, *GJ-tmp*, and *GJ-trp*) and five populations of indica (*XI-1A*, *XI-1B*, *XI-2*, *XI-3*, and *XI-adm*). (**B**) Kinship heat map of 469 rice. The indica subpopulation in the lower left corner and the japonica subpopulation in the upper right corner.

PCA and relatedness (KI) were used to quantify the population structure of the 469 germplasm varieties. PCA showed that the 469 germplasm varieties were distinguished into two populations, the indica subpopulation and the japonica subpopulation, which were refined to reduce false positives and divided further into nine populations, four japonica groups and five indica groups (Figure 2A). Kinship analysis showed convergence in two directions, with a distinct division into indica and japonica subpopulations, consistent with the PCA results (Figure 2B).

### 2.3. ETN-Related QTL Detected by GWAS

To detect genome-wide associated loci with ETNs, GWAS was performed utilizing 2,801,841 high-quality SNPs and phenotypic data. The GWAS was conducted using a mixed linear model in the EMMAX program. After applying the Bonferroni correction, the *p*-values were estimated to have suggestive and significant thresholds of 1 × 10^−5^ based on the independent markers (Table S3). A total of 50 ETN-related QTL intervals were obtained, distributed on chromosomes 1, 2, 3, 4, 5, 6, 7, 8, 10, 11 and 12. 24 QTLs were detected in 2021. 26 QTLs were detected in 2022, among them, 13 QTLs were detected under low nitrogen conditions, which were named *qLETN2-1*, *qLETN2-4*, *qLETN2-5*, *qLETN2-6*, *qLETN2-7*, *qLETN3-1*, *qLETN4-1*, *qLETN4-3*, *qLETN5-1*, *qLETN5-1*, *qLETN6-1*, *qLETN7-1*, and *qLETN8-1*. Six QTLs were detected under high nitrogen conditions, which were named *qHETN1-2*, *qHETN1-3*, *qHETN2-8*, *qHETN8-2*, *qHETN10-1* and *qHETN12-2*. Seven QTLs were detected under NRI conditions, which were named *qNRI2-4*, *qNRI2-5*, *qNRI2-6*, *NRI2-7*, *qNRI5-3*, *qNRI7-2* and *qNRI9-1* (Figure 2B, Table S4). In this study, we screened QTLs that were co-detected under different treatments or co-detected over 2 years. A total of 28 QTLs were screened, and distributed on 10 chromosomes (Table 2).

### 2.4. ETN-Related QTL Co-Localized with Previously Reported Rice Tillering Genes

**Figure 3:**
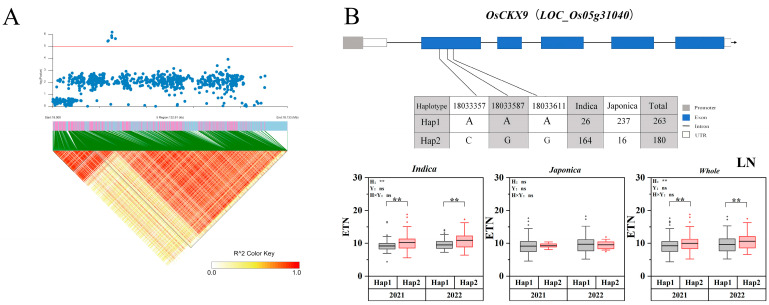
Associated region and haplotype analysis of *OsCKX9*. (**A**) Regional Manhattan plots and linkage distribution (LD) heatmap of *OsCKX9*. (**B**) Gene structure and haplotype analysis of *OsCKX9*. LD blocks indicated ± 200 kb. LN: Low nitrogen; ETN: Effective tillering number. The box plot describes the ETN distribution of a subgroup with a haplotype. Significant differences in two-way ANOVA (** *p* < 0.01, ** indicates significant difference in average ETN among 469 accessions at *p* < 0.01, ns: no significant. H: Haplotypes, Y: Years, H × Y: Haplotypes × Years).

In this study, two genes co-localized with tillering-related genes from previous studies, including *NAL1* and *OsCKX9*. *OsCKX9* is a cytokinin oxidase/dehydrogenase (CKXs), which belongs to a group of enzymes that regulate oxidative cleavage to maintain cytokinin homeostasis. The *OsCKX9* double mutant increased the number of tillers and the number of panicles per plant [32]. The LD of *OsCKX9* is shown in Figure 3A. *OsCKX9* was located 1.1 kb upstream of the peak SNP (*rs5_18033587*) of *qLETN5-1*. Additionally, three nonsynonymous SNPs were identified in the CDS region, which contained two haplotypes of *OsCKX9*. Most of the indica rice populations contained haplotype 2. Furthermore, the ETN of haplotype 2 were higher than those of haplotype 1 throughout the indica and whole populations (Figure 3B).

**Figure 4:**
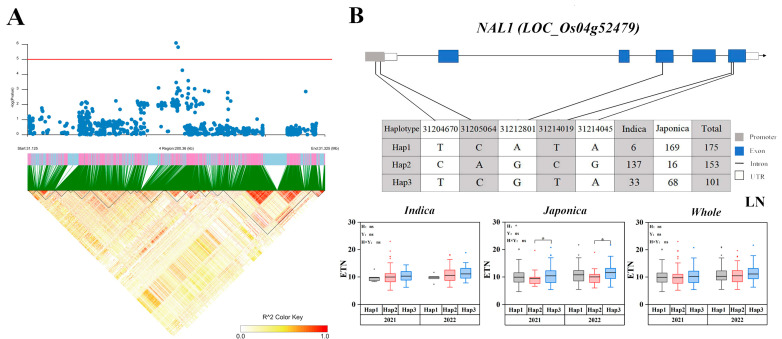
Associated region and haplotype analysis of *NAL1*. (**A**) Regional Manhattan plots and linkage distribution (LD) heatmap of *NAL1*. (**B**) Gene structure and haplotype analysis of *NAL1*. LD blocks indicated ± 200 kb. LN: Low nitrogen; ETN: Effective tillering number. The box plot describes the ETN distribution of a subgroup with a haplotype. Significant differences in two-way ANOVA (* *p* < 0.05, * indicates significant difference in average ETN among 469 accessions at *p* < 0.05, ns: no significant. H: Haplotypes, Y: Years, H × Y: Haplotypes × Years).

*NAL1*, which was initially cloned from a loss-of-function mutant showing narrow leaves and increased tiller number, was identified [33]. The LD of *NAL1* is shown in Figure 4A. *NAL1* was located approximately 21.06 kb upstream of the peak SNP (*rs4_31224587*) of *qLETN4-3*. Five SNPs located in the promoter region and coding sequence (CDS) region of *NAL1* formed three haplotypes. Varieties containing haplotype 3 had a significantly higher ETN than those of haplotype 2 within the japonica rice population (Figure 4B).

### 2.5. Candidate Gene Haplotype Analysis

**Figure 5:**
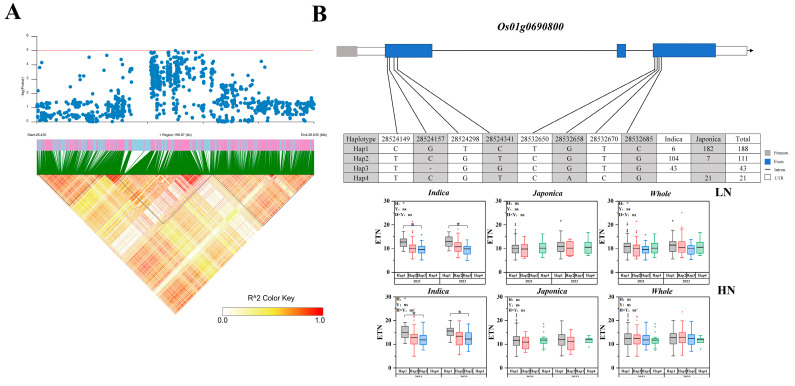
Associated region and haplotype analysis of *Os01g0690800*. (**A**) Regional Manhattan plots and linkage distribution (LD) heatmap of *Os01g0690800*. (**B**) Gene structure and haplotype analysis of *Os01t0690800*. LD blocks indicated ± 200 kb. LN: Low nitrogen; HN: High nitrogen; ETN: Effective tillering number. The box plot describes the ETN distribution of a subgroup with a haplotype. Significant differences in two-way ANOVA (* *p* < 0.05, * indicates significant difference in average ETN among 469 accessions at *p* < 0.05, ns: no significant. H: Haplotypes, Y: Years, H × Y: Haplotypes × Years).

*qLETNL1-2*, *qHETNL1-2*, *qLETNL2-4*, *qNRI2-4*, *qLETNL2-5*, *qNRI2-5*, *qLETNL2-6*, *qNRI2-6*, and *qLETNL4-3* were detected under multiple conditions and years and were used as the main intervals for candidate gene mining. Haplotype analysis showed significant differences among the haplotypes of five genes. Haplotype analysis was performed on five candidate genes. *Os01g0690800*, a gene encoding a protein containing a protein kinase domain, was detected in low nitrogen *qLETNL1-2* and high nitrogen *qHETN1-2*. The LD of *Os01g0690800* is shown in Figure 5A. Eight nonsynonymous SNPs were identified in the CDS region, constituting four haplotypes of *Os01g0690800*. Under both low and high nitrogen conditions, the ETN of haplotype 1 was significantly higher than that of haplotype 3 in the indica subpopulation (Figure 5B).

**Figure 6:**
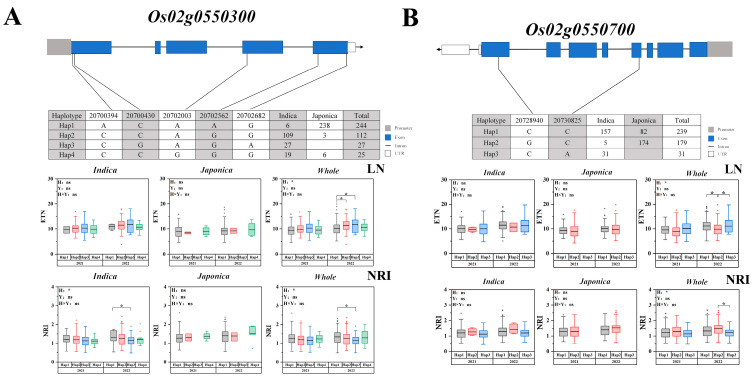
Associated region and haplotype analysis of *Os02g0550300* and *Os02g0550700* (**A**) Gene structure and haplotype analysis of *Os02g0550300*. (**B**) Gene structure and haplotype analysis of *Os02g0550700*. LD blocks indicated ± 200 kb. LN: Low nitrogen; NRI: Ratio of high to low nitrogen. The box plot describes the ETN distribution of a subgroup with a haplotype. Significant differences in two-way ANOVA (* *p* < 0.05, * indicates significant difference in average ETN among 469 accessions at *p* < 0.05, ns: no significant. H: Haplotypes, Y: Years, H × Y: Haplotypes × Years).

In this study, the same area (chromosome 2 20316453-20806338) was localized under different conditions. There are haplotype differences in *Os02g0550300* and *Os02g0550700*. The LD of *Os02g0550300* and *Os02g0550700*, is shown in Figure S3. Five nonsynonymous SNPs were identified in the CDS region, resulting in four haplotypes of *Os02g0550300*. The ETN of haplotype 1 was lower than that of the other haplotypes in the whole population under low nitrogen conditions (Figure 6A). The NRI of haplotype 1 was higher than that of the others in the whole population. *Os02g0550700* is similar to a nucleolar autoantigen-like protein. Two nonsynonymous SNPs were identified in the CDS region, consisting of three haplotypes of *Os02g0550700*. Haplotype 1 was the major haplotype of the indica population, haplotype 2 being the major haplotype of the japonica population, while haplotype 3 was only observed in the indica population. Under low nitrogen conditions, the ETN of haplotype 3 was significantly higher than that of haplotype 2 in the whole population, the NRI of haplotype 3 was less than that of haplotype 2 in the whole populations (Figure 6B). Thus, haplotype 3 was a favorable allele of *Os02g0550700*.

**Figure 7:**
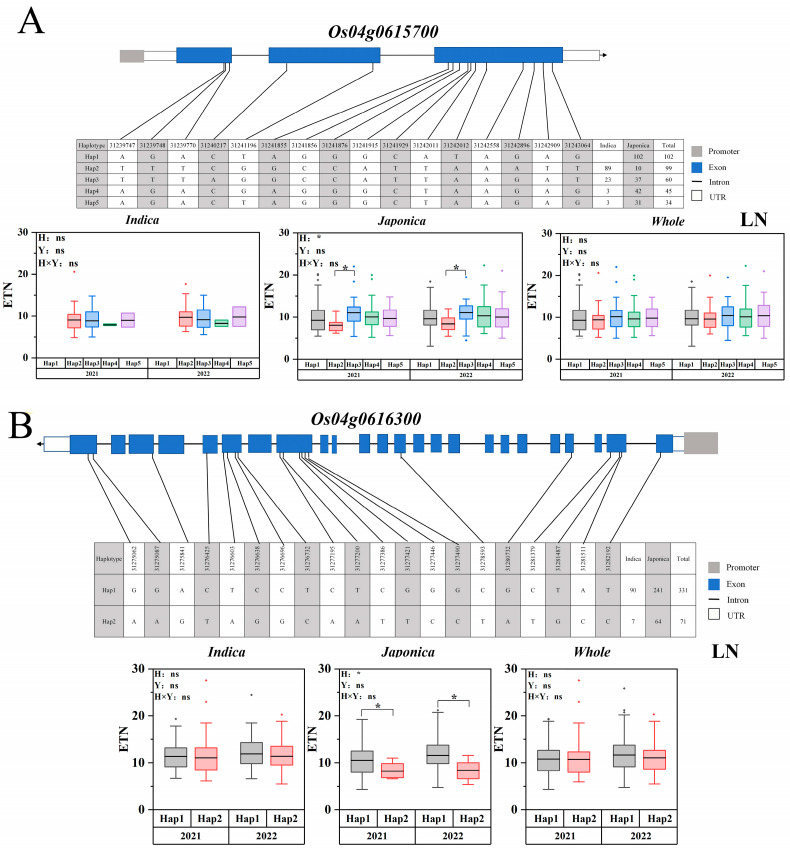
Associated region and haplotype analysis of *Os04g0615700* and *Os04t06163000*. (**A**) Gene structure and haplotype analysis of *Os04g0615700*. (**B**) Gene structure and haplotype analysis of *Os04t06163000*. LD blocks indicated ± 200 kb. LN: Low nitrogen. The box plot describes the ETN distribution of a subgroup with a haplotype. Significant differences in two-way ANOVA (* *p* < 0.05, * indicates significant difference in average ETN among 469 accessions at *p* < 0.05, ns: no significant. H: Haplotypes, Y: Years, H × Y: Haplotypes × Years).

In this study, two candidate genes of *Os04g0615700* and *Os04g06163000* were identified on chromosome 4. The LD of *Os04g0615700* and *Os04g06163000* is shown in Figure S4. Within the five haplotypes of *Os04g0615700*, a total of 16 nonsynonymous SNPs were identified in the CDS region. Significant differences were identified between the ETN of haplotype 2 and the ETN of haplotype 3 in the japonica population (Figure 7A). Two haplotypes of *Os04g06163000* included 20 nonsynonymous SNPs in the CDS region. The ETN of haplotype 1 was significantly higher than that of haplotype 2 in the japonica population (Figure 7B).

### 2.6. Temporal Expression Patterns of ETN-Related Genes

**Figure 8:**
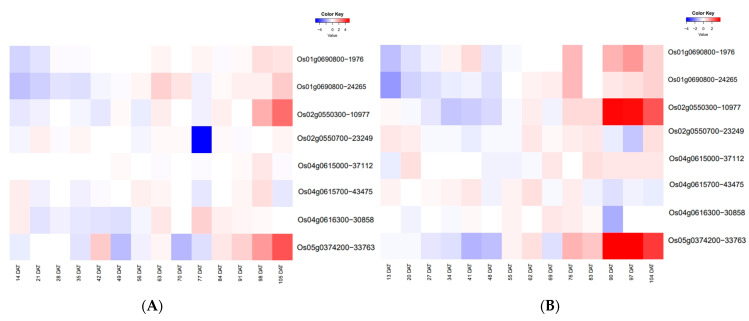
Temporal expression pattern of efficient total nitrogen (ETN)-associated genes during the whole growth period. Expression data in root (**A**) at 12:00 and (**B**) 00:00 have been downloaded from the RiceXPro website (https://ricexpro.dna.affrc.go.jp/, accessed on 7 November 2022). The heatmaps represent hierarchical clustering of relative expression levels of 12 candidate genes on different days after transplanting (DAT). The scale for relative expression levels (after normalization by z-score) is denoted by color bars, with red representing high expression levels, white representing medium expression, and blue representing low expression.

Tiller developmental regulation may exhibit specific spatio-temporal patterns whose expression in the root system at 00:00 (R0) and 12:00 (R12) is stable and high from 20 d after transplanting (DAT) to 48 DAT [34]. Temporal expression patterns of the above five genes and two reported genes were analyzed using gene expression data from the RiceXPro website (https://ricexpro.dna.affrc.go.jp/, accessed on 7 November 2022) (Figure 8). *Os01g0690800*, *Os02g0550300*, *Os02g0550700*, *NAL1*, *Os04g0615700* and *OsCKX9* in R0 and *Os01g0690800*, *Os02g0550700*, *NAL1*, *Os04g0615700*, and *Os04g06163000* in R12 were found to have higher expression between 21 DAT and 49 DAT, which was consistent with the full fertility dynamic pattern of the rapid increase in tiller number starting from 21 DAT [35].

## 3. Discussion

### 3.1. Phenotypic Variation in ETN of Rice

In this study, the phenotypic analysis of 469 rice varieties showed that the frequency distribution of the phenotype was normally distributed (Table S5). Combining the two-year data, the ETN of each population under high nitrogen treatment was higher than that under low nitrogen treatment. Meanwhile, the ETN of the indica rice population was significantly higher than that of the japonica rice population, regardless of high or low nitrogen, which was similar to the results of Ren et al. [36]. The results of this study suggest that germplasm resources exhibit significant genetic variation in agronomic traits and dissect effective tiller genetic differences between the indica and japonica rice populations [37,38].

### 3.2. Comparisons of QTL Detected in This Study with Previously Reported Genes

Unlike previous mapping studies, which have mostly been based on classical QTL mapping [6,32,39], GWAS can successfully map a large number of potential loci. This study was conducted by GWAS using three effective tiller traits (low nitrogen, high nitrogen, and NRI) and 2,801,841 SNPs from a natural population of 469 rice varieties. In this study, *qLETN3-1* was coincident with the mapping results of Zhao et al. [40], while *qLETN4-1* and *qLETN4-3* were coincident with the QTL associated with effective tillering identified by Lanceras et al. [41]. The genes associated with tillering number *NAL1* were located in *qLETN4-3*. *NAL1* contributes to leaf size and traits [42], transport of growth hormone [43], vascular bundle [44], rice yield traits [45]. This study also detected a known gene, *OsCKX9*, which is associated with an effective panicle number. As the main response gene of SL, the *OsCKX9* mutant and overexpression transgenic plants have exhibited a significant increase in tiller number [32]. These results provide a reference for further study of gene verification and molecular marker-assisted selection.

### 3.3. Candidate Gene Identification for Important QTLs

Effective tillering is a complex trait controlled by many genes, while several recent studies have identified genes involved in the mechanism through which nitrogen affects rice tillering [46], including *MH63* [47], *OsAAP4* [48], and *OsNPF7.3* [49]. The haplotypes of seven ETN-related genes were analyzed. These seven genes showed different haplotype patterns in indica, japonica, and the whole population. The haplotypes of some new genes identified in this study performed well under different treatments. Haplotype 1 of *Os01g0690800* performed well in indica subsets under low nitrogen and NRI conditions, and haplotype 3 of *Os02g0550300* performed well in total populations under low nitrogen and NRI conditions. Haplotype 2 of *Os02g0550700* performed well in the total population. These candidate genes were combined with gene annotation to identify protein kinases similar to *Os01g0690800*. Studies have shown that protein kinases are able to regulate tillering. For example, the *OsKAPP* protein kinase regulates growth hormone levels and thus affects rice tillering [50]. To our knowledge, there are no reports on the breeding value of these five genes. These results have inspired the further exploration of favorable alleles in response to low-nitrogen ETN breeding using natural resources.

The prevailing spatio-temporal expression patterns of five candidate genes and two known genes in R0 and R12, namely *NAL1*, *OsCKX9*, *Os01g0690800*, *Os02g0550300*, *Os02g0550700*, *Os04g0615700*, and *Os04g06163000*, with relatively high expression from 21 DAT to 49 DAT. This expression pattern coincides with the dynamic pattern of tiller number during the entire growth period, where tiller number increased rapidly from 21 DAT and peaked at 49 DAT [35]. Using this expression pattern, convincing candidate genes were thereby identified.

## 4. Materials and Methods

### 4.1. Plant Materials

The materials used in this study were re-sequenced germplasm varieties from the 3K RGP (https://snp-seek.irri.org/, accessed on 4 May 2020). These rice varieties possess a rich phenotype with globally diverse genetic backgrounds. This experiment selected 469 varieties with similar heading stage, including 202 indica rice and 267 japonica rice. Detailed information of the 469 varieties, including their geographical origin, is described in Table S1.

### 4.2. Phenotype Determination

The field trial was carried out at Shenyang Agricultural University (41°48′ N, 123°34′ E). Two nitrogen levels were tested in the field trial: 60 kg/hm^2^ for low nitrogen conditions (LN) and 180 kg/hm^2^ for high nitrogen conditions (HN). Additionally, a water barrier was placed between the plots. The plant spacing was 13.3 cm × 30 cm, with two plants being planted per hole after transplanting, while one row of each variety was planted in a 1 m row length, with routine field management. An effective tiller survey was conducted at maturity stage. Border plants were removed, and six plants from the middle were randomly selected, evaluation of ETN with effective tiller being defined as the one bearing more than 10 seeds at maturity stage, and the mean value was used for further analyses.

### 4.3. Phenotypic Data Analysis

Using IBM SPSS Statistics 26.0 software SPSS, origin and R 4.2.1 language statistical analysis software, the phenotypic values of the measured traits were statistically analyzed: a. test the accuracy of the values, delete the abnormal values; b. statistical distribution of phenotypic values; c. The differences of phenotypic values of haplotypes of each gene in indica rice subgroup, japonica rice subgroup and total population were analyzed. Broad-sense heritability (*H*^2^) was calculated based on the following formular: *H*^2^ = *VG/*(*VG + VE*), where *VG* and *VE* are genetic and environmental variances (Table S6).

### 4.4. Genotyping Data Analysis

The raw genotype data of the 469 accessions were obtained from the 3K RGP Rice SNP-Seek Database website (https://snp-seek.irri.org/, accessed on 4 May 2020). Using the linkage disequilibrium (LD) pruning tool of PLINK 1.9, we obtained independent SNPs with a genotype missing rate ≤ 20% and minor allele frequency ≥1% according to the settings “indep-pairwise 50 10 0.5” [51]. The remaining high-quality SNPs were evenly distributed on chromosomes and these SNP markers were used for genome-wide association analysis.

Kinship matrix is a call to the IBS/IBD calculation. IBS scores are calculated based on whether two individuals have the same genotype data at the same locus, and the scores range from 0 to 2, with higher values indicating a greater degree of similarity between the two individuals. The IBS score between two individuals is calculated using the following formula: IBS = (2 × AA + AB)/(2 × n), where AA represents the number of two individuals with exactly the same genotype data at a certain point, AB represents the number of two individuals with only one genotype difference in genotype data at a certain point, and n represents the number of loci that two individuals consider when calculating the IBS score [52]. Principal component analysis (PCA) and kinship analysis were performed using PLINK and plotted with R 4.2.1 “ggplot2” package and “Cmplot” package.

### 4.5. Genome-Wide Association Analysis

To minimize the effect of false positives, 4.8M SNPs with the missing rate ≥ 20% and MAF ≤ 1% were removed by PLINK 1.9 software. Finally, a total of 2,801,841 SNPs in the 469 rice accessions and the average ETN of each accession were used to carry out GWAS. The Efficient Mixed-Model Association eXpedited (EMMAX) was used to perform GWAS based on the SNP genotypes and ETN on plants inoculated with low and high nitrogen levels using a mixed linear model linear (MLM), principal component analysis (PCA), and kinship. The threshold for genome-wide significance was determined by Bonferroni correction (that is, corrected *p* = 0.05/n, in which n is the number of independent SNPs across the genome [53]. The Manhattan and quantile–quantile (Q–Q)plots were generated using the R package “CMplot” (Supplementary Figure S2, Supplementary Figure S5), the chromosomal region where each QTL was located was determined when the linkage disequilibrium (LD) dropped to half its maximum.

### 4.6. Candidate Gene Haplotype Analysis

The linkage disequilibrium (LD) is calculated as follows: If there are two loci (A and B), and the alleles are A, a, B, and b, which correspond to frequencies f(A), f(a), f(B), and f(b) in the population. There are four haplotypes AB, Ab, aB, and ab at the two loci, corresponding to frequencies f(AB), f(Ab), f(aB), and f(ab). Calculation: Dab = f(AB) − f(A) × f(B), when Dab = 0, it is in the chain equilibrium state; When Dab ≠ 0, it is in a chain imbalance. LD metric: When Dab > 0, |D′| = (Dab)^2^/min(f(AB), f(ab)); When Dab < 0,|D′| = (Dab)^2^/min(f(Ab), f(aB)); r^2^ = (Dab)^2^/(f(A)f(a)f(B) × f(b)). D′ = 0, r^2^ = 0 is in complete chain equilibrium; D′ = 1, r^2^ = 1 is in a state of complete chain disequilibrium. The higher the scale from 0 to 1, the higher the LD, and if the two loci are linked, the degree of linkage is also stronger [54]. The LD heatmap around the peak in GWAS was constructed using “LD BlockShow”.

According to LD decay, the region 200 kb upstream and downstream of a significant SNP was classified as an LD block. An LD block containing more than two significant SNPs was defined as a QTL [55]. In each QTL, the SNP with minimum *p*-value was considered as the lead SNP [56]. Based on GWAS, QTLs with multi-year co-localization or different processing co-localization were screened, with candidate genes being mined for target traits responding to effective tillering in rice.

Haplotype analysis was performed on all genes in the QTL associated with effective tillering under different nitrogen levels during 2021 and 2022. The procedure was as follows: First, all gene numbers and annotation information in each important QTL interval were retrieved from the Rice Annotation Project Database (RAP-DB) (http://rapdb.dna.affrc.go.jp/, accessed on 28 June 2022), before all nonsynonymous mutation SNPs of all genes and SNPs in the promoter region were extracted from the Rice SNP-Seek Database website of 3K RGP. The above gene screening method was used to eliminate the lower quality SNPs, with the remaining high-quality SNPs being used for haplotype analysis. Haplotype analysis was performed on all genes using nonsynonymous mutant SNPs. Genes with no significant differences in phenotypic values between different haplotypes were detected before haplotype analysis was performed using SNPs in the promoter region. Haplotype analysis was then performed based on nonsynonymous mutations and SNPs in the promoter region (1 kb before the ATG). The SNP in the promoter region was used for haplotype analysis. Multiple comparisons of phenotypic values of different haplotypes (≥15 varieties) were performed to screen genes with significant differences between phenotypic values of different haplotypes and to predict possible candidate genes in combination with the gene annotation. Subsequently, the RiceXPro website (http://ricexpro.dna.affrc.go.jp/, accessed on 7 November 2022) was used to accurately identify candidate genes.

## 5. Conclusions

The study utilized phenotypic data from 469 effective tillers from natural populations and 2,801,841 SNPs for GWAS analysis. Among the 28 QTL intervals identified in this study, haplotype analysis was performed on genes that were repeatedly located within QTLs over multiple years or with different treatments. A total of seven genes (*NAL1*, *OsCKX9*, *Os01g0690800*, *Os02g0550300*, *Os02g0550700*, *Os04g0615700*, and *Os04g06163000*) were pinpointed in these QTL regions, and were considered the most likely candidate genes. Cluster analysis was employed to analyze the spatial and temporal expression patterns of these seven candidate genes, leading to the identification of convincing candidate genes. The results obtained provide favorable information for the use of marker selection to control effective tillering in rice for yield improvement. However, further research is needed to understand the regulatory mechanism of theses candidate genes that contribute to low nitrogen tolerance in rice.

## Figures and Tables

**Table 1:** Descriptive statistics of effective tillers under different nitrogen levels in 2021 and 2022.

Year	Nitrogen	Subpopulation	Average	SD	Skewness	Kurtosis	Min	Max	CV%
2021	LN	Indica	10.60	3.33	1.41	1.11	5.20	22.00	31.44
		Japonica	8.24	2.47	1.29	0.94	3.33	18.40	29.92
		Whole	9.35	2.42	0.74	0.78	3.00	17.25	29.07
	HN	Indica	12.05	3.76	2.37	1.31	5.80	26.80	31.24
		Japonica	9.96	3.00	0.77	0.77	4.25	20.00	30.12
		Whole	11.14	3.60	2.39	1.20	4.25	26.80	32.30
2022	LN	Indica	11.21	3.36	4.59	1.58	5.17	27.50	29.99
		Japonica	10.12	3.27	0.10	0.80	4.33	19.25	32.30
		Whole	10.63	3.35	2.46	1.15	4.33	27.50	31.52
	HN	Indica	13.48	3.91	3.09	1.14	6.20	33.00	29.01
		Japonica	11.96	4.20	1.08	1.02	4.83	26.67	35.15
		Whole	12.70	4.13	1.72	0.98	4.83	33.00	32.53

**Table 2:** Genetic loci associated with effective tiller number were studied by genome-wide association.

Chr	Start	End	Year	Subpopulation	QTL	*p*-Value	Candidate/Known Gene
1	28312507	28635235	2021	Indica	*qLETN1-2*	9.7639 × 10^−6^	*Os01g0690800*
			2021	Indica	*qHETN1-2*	4.96655 × 10^−6^	
			2022	Indica, Whole	*qHETN1-2*	3.05122 × 10^−6^	
2	1925017	2258654	2021	Indica	*qLETN2-1*	3.77201 × 10^−6^	
				Indica, Whole	*qHETN2-1*, *qHETN2-3*	8.0282 × 10^−7^	
			2022	Whole	*qLETN2-1*	7.92919 × 10^−6^	
2	20316453	20806338	2022	Indica, Whole	*qLETN2-4*, *qLETN2-5*, *qLETN2-6*, *qLETN2-7*	8.01316 × 10^−7^	*Os02t0550300* *Os02t0550700*
				Indica, Whole	*qNRI2-4*, *qNRI2-5*, *qNRI2-6*	5.38504 × 10^−7^	
3	28202203	28408332	2021	Indica	*qLETN3-1*	1.25578 × 10^−6^	
			2022	Indica	*qLETN3-1*	7.8412 × 10^−6^	
4	31125957	31325965	2021	Japonica, Whole	*qLETN4-3*	4.1416 × 10^−6^	*NAL1* [30] *Os04g0615700* *Os04t0616300*
			2022	Japonica, Whole	*qLETN4-3*	3.52586 × 10^−6^	
5	18033644	18260106	2021	Indica	*qLETN5-1*	3.84225 × 10^−6^	*OsCKX9* [31]
			2022	Indica	*qLETN5-1*	5.65649 × 10^−7^	
5	20672878	20956221	2021	Whole	*qNRI5-3*	8.01541 × 10^−6^	
			2022	Whole	*qNRI5-3*	8.29562 × 10^−6^	
7	9167844	9368439	2021	Japonica	*qLETN7-1*	4.6369 × 10^−6^	
			2022	Japonica	*qLETN7-1*	7.8421 × 10^−6^	
7	27836562	28048531	2021	Indica	*qNRI7-2*	6.56564 × 10^−6^	
			2022	Indica	*qNRI7-2*	5.6232 × 10^−6^	
8	20505376	20723565	2021	Whole	*qLETN8-1*	8.65652 × 10^−6^	
			2022	Whole	*qLETN8-1*	7.51026 × 10^−6^	

## Data Availability

The data supporting the findings of this study are available within the article and its Supplementary Materials.

## Supplementary Materials

The supporting information can be downloaded at: https://www.mdpi.com/article/10.3390/ijms25052969/s1.
