# Supplementary material for: Genome-Wide Association Analysis of Effective Tillers in Rice under Different Nitrogen Gradients
Source: Int J Mol Sci. 2024 Mar 4;25(5):2969. doi: 10.3390/ijms25052969 (PMC10932320; doi:10.3390/ijms25052969)
Supplement: Supplementary file 1 [file ijms-25-02969-s001.zip › supplementary files/Figure S1-S5.pdf]

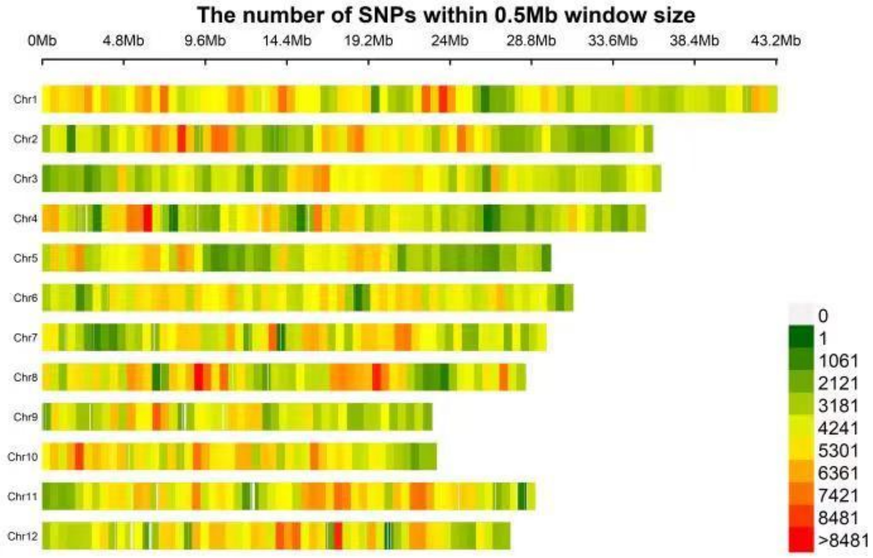

Figure S1. SNP density map for rice chromosomes.

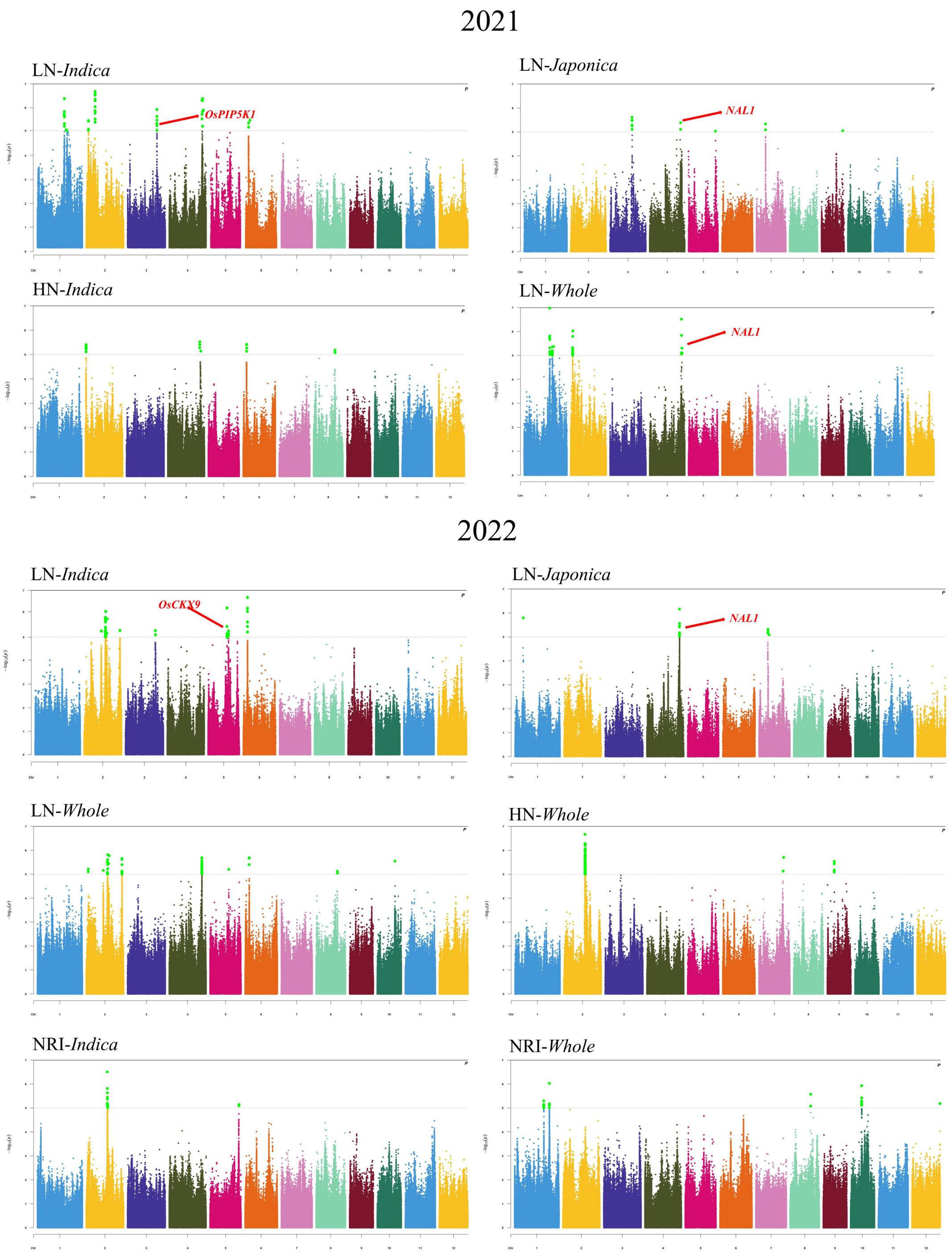

Figure S2. Manhattan map of genome-wide association analysis of rice ETN at different nitrogen levels

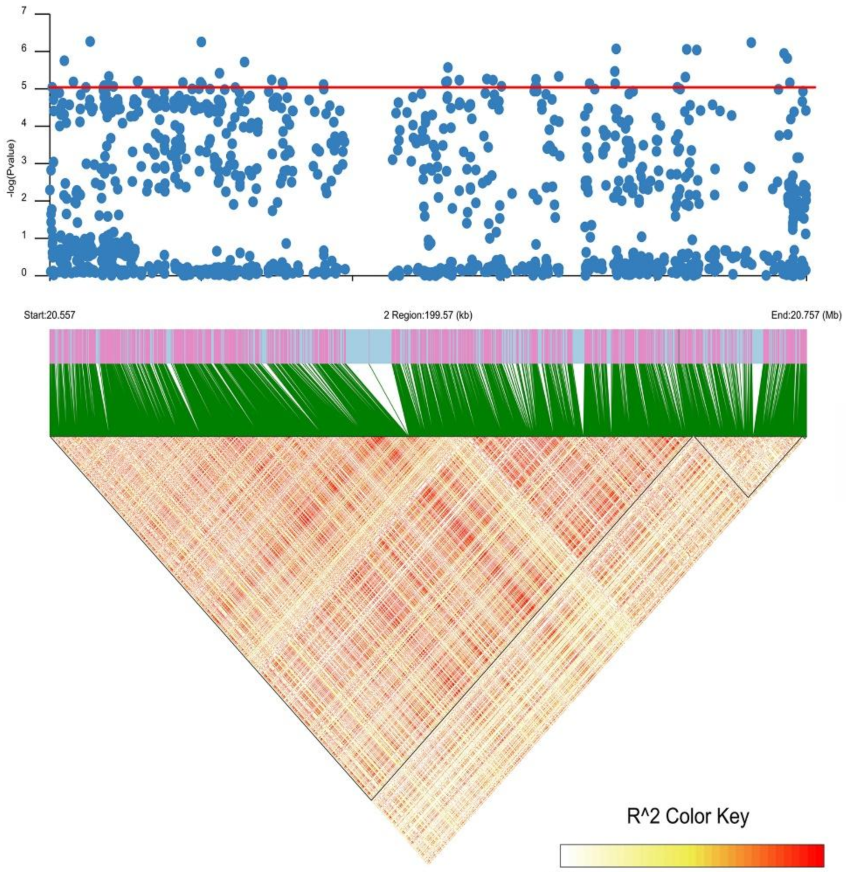

Figure S3. Regional Manhattan plots and linkage distribution (LD) heatmap of *Os02g0550300 and Os02g0550700*.

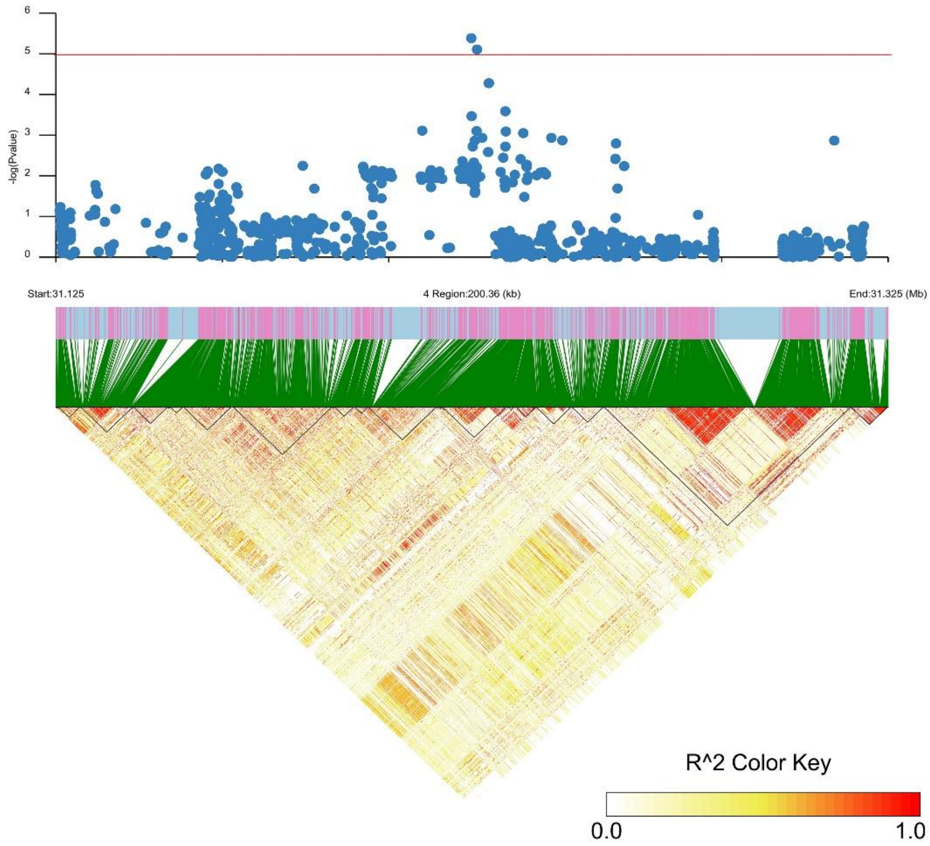

Figure S4. Regional Manhattan plots and linkage distribution (LD) heatmap of *Os04g0615700* and *Os04t06163000*.

2021

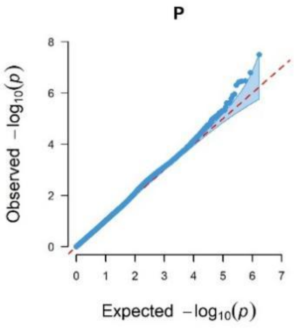

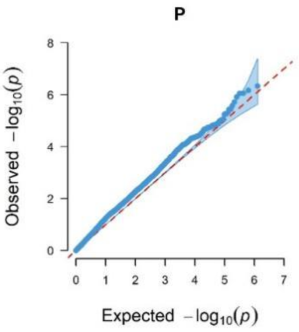

LN-*Indica* LN-*Japonica*

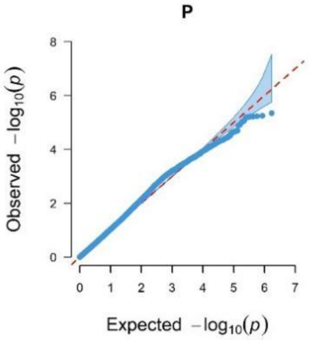

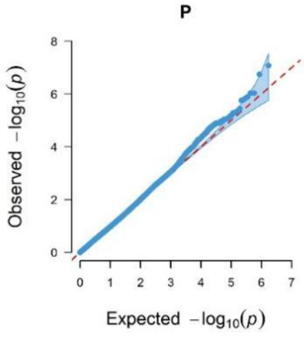

HN-*Indica* LN-*Whole*

2022

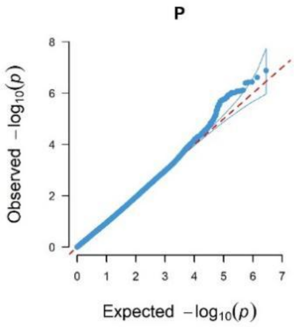

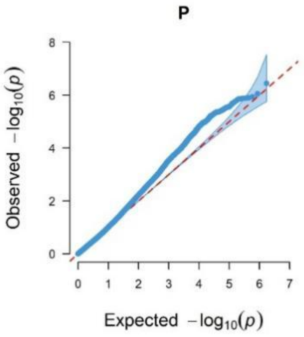

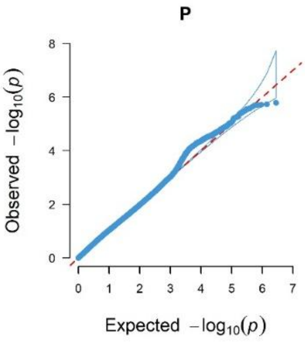

LN-*Indica* LN-*japonica* LN-*Whole*

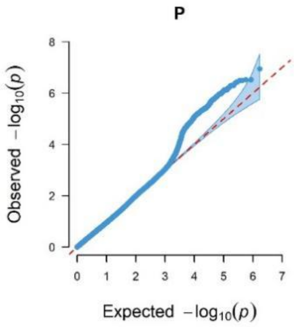

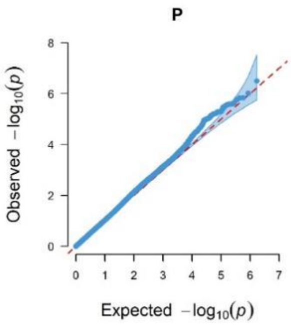

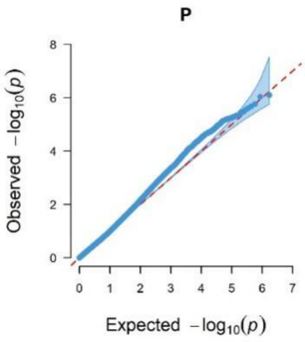

HN-*Whole* NRI-*Indica* NRI-*Whole*

Figure S5. Quantile–quantile plots for GWAS of effective tillering number
